# Targeted Therapies for Perihilar Cholangiocarcinoma

**DOI:** 10.3390/cancers14071789

**Published:** 2022-03-31

**Authors:** Simon Gray, Angela Lamarca, Julien Edeline, Heinz-Josef Klümpen, Richard A. Hubner, Mairéad G. McNamara, Juan W. Valle

**Affiliations:** 1 Department of Medical Oncology, The Christie NHS Foundation Trust, Wilmslow Rd, Manchester M20 4BX, UK; simon.gray6@nhs.net (S.G.); angela.lamarca@nhs.net (A.L.); richard.hubner@nhs.net (R.A.H.); mairead.mcnamara@nhs.net (M.G.M.); 2 Division of Cancer Sciences, University of Manchester, Oxford Rd, Manchester M13 9PL, UK; 3 Centre Eugène Marquis, Av. de la Bataille Flandres Dunkerque-CS 44229, CEDEX, 35042 Rennes, France; j.edeline@rennes.unicancer.fr; 4 Department of Medical Oncology, Amsterdam University Medical Center, P.O. Box 7057, 1081 HV Amsterdam, The Netherlands; h.klumpen@amsterdamumc.nl

**Keywords:** perihilar, cholangiocarcinoma, pCCA, extrahepatic, targeted therapy, biliary tract cancer

## Abstract

**Simple Summary:**

Perihilar cholangiocarcinoma is a type of biliary tract cancer with a poor prognosis. There is growing interest in treatments targeting specific molecular changes within these cancers, and a broad range of these treatments (targeted therapies) is currently in development. This article reviews knowledge of the molecular changes within cholangiocarcinoma, which often group together to form so-called molecular subtypes, and reviews the targeted therapies used to exploit these molecular changes to improve outcomes for patients. Biliary tract cancer subtypes differ in their patterns of molecular alterations; therefore, targeted therapies are not equally applicable to all subtypes. This article examines the relevance of targeted therapies within biliary tract cancer to patients with perihilar cholangiocarcinoma.

**Abstract:**

Perihilar cholangiocarcinoma (pCCA) is the anatomical sub-group of biliary tract cancer (BTC) arising between the second-order intrahepatic bile ducts and the cystic duct. Together with distal and intrahepatic cholangiocarcinoma (dCCA and iCCA; originating distal to, and proximal to this, respectively), gallbladder cancer (GBC) and ampulla of Vater carcinoma (AVC), these clinicopathologically and molecularly distinct entities comprise biliary tract cancer (BTC). Most pCCAs are unresectable at diagnosis, and for those with resectable disease, surgery is extensive, and recurrence is common. Therefore, the majority of patients with pCCA will require systemic treatment for advanced disease. The prognosis with cytotoxic chemotherapy remains poor, driving interest in therapies targeted to the molecular nature of a given patient’s cancer. In recent years, the search for efficacious targeted therapies has been fuelled both by whole-genome and epigenomic studies, looking to uncover the molecular landscape of CCA, and by specifically testing for aberrations where established therapies exist in other indications. This review aims to provide a focus on the current molecular characterisation of pCCA, targeted therapies applicable to pCCA, and future directions in applying personalised medicine to this difficult-to-treat malignancy.

## 1. Introduction

Biliary tract cancer (BTC) is a heterogeneous entity comprising cholangiocarcinoma (CCA), gallbladder cancer (GBC) and ampulla of Vater carcinoma (AVC). Cholangiocarcinoma is subdivided into intrahepatic, perihilar and distal CCA (iCCA, pCCA and dCCA, respectively) [1]. The term extrahepatic CCA (eCCA) is no longer recommended, because pCCA and dCCA are now considered distinct clinicopathologic entities [2], although the term remains prevalent and limits the direct application of many study results to patients with pCCA. Mixed hepatocellular carcinoma (HCC)–CCA shares features of both HCC and iCCA, and is considered distinct from BTC [3]. Cholangiocarcinoma represents the second most common primary liver malignancy after HCC, representing 3% of gastrointestinal cancers [4]. Due to a lack of clinical symptoms in its early stages, CCA presents as unresectable (locally advanced or metastatic) in approximately 75% of patients. Surgical resections are typically extensive, with significant associated morbidity and mortality: the median recurrence-free survival (RFS) of resected patients is approximately 2 to 3 years, and may vary depending on anatomical sub-group [5,6,7]. Following resection, the current standard-of-care adjuvant treatment is capecitabine, based on the BILCAP study in which capecitabine conferred a median overall survival (mOS) of 51.1 months (95% CI 34.6–59.1) and a median RFS of 24.4 months (95% CI 18.6–35.9) on intention-to-treat analysis. Patients with pCCA appeared to derive little benefit from capecitabine (HR 1.08; 95% CI 0.68–1.71), although the study was not powered for such subgroup analysis [8]. For patients with BTC who present with unresectable, metastatic or recurrent disease, first-line standard-of-care cisplatin and gemcitabine (Cis/Gem) provides a median progression-free survival (mPFS) of 8.0 months and a mOS of 11.7 months, as reported in the landmark ABC-02 study [9]; its efficacy was subsequently confirmed in a Japanese population in the BT22 study [10]. The recently published TOPAZ-1 study suggests a benefit with the addition of the programmed death receptor ligand 1 (PD-L1) antibody durvalumab to Cis/Gem in molecularly unselected patients [11], and is discussed further in Section 3.4. Following the results of the ABC-06 study, an option for second-line chemotherapy for BTC is 5-fluorouracil, folinic acid and oxaliplatin (FOLFOX), which conferred a mOS of 6.2 months (95% CI 5.4–7.6), compared with 5.3 months (95% CI 4.1–5.8; hazard ratio [HR] 0.69; 95% CI 0.50–0.97; *p* = 0.031) for active symptom control alone with improved 6- and 12-month survival rates [12]. Following the NIFTY study, a further second-line option may be 5-fluorouracil/liposomal irinotecan, associated with a mPFS of 7.1 months (95% CI 3.6–8.8) and a mOS of 8.6 months (95% CI 5.4–10.5) [13]. The modest benefit with cytotoxic chemotherapy regimens has been a driver towards developing targeted therapies in CCA.

Anatomically, pCCA is defined as CCA arising between the second-order bile ducts (proximal to which is iCCA) and the cystic duct (distal to which is dCCA) [4]. The incidence of all subtypes of CCA shows considerable geographic variation, ranging from 85 cases per 100,000 people in Thailand to 0.4 per 100,000 in Canada [2]. Assessments of trends in the relative incidence of anatomical CCA subtypes have been hampered by the omission of a separate diagnostic code for pCCA in previous iterations of both the International Classification of Diseases (ICD) and the ICD-Oncology (ICD-O) [2,14]. A common feature among many risk factors for CCA is chronic inflammation of the biliary tree; infection by the liver flukes *Opisothorchis viverrini* and *Clonorchis sinensis* account for some of the highest rates of CCA, seen in endemic areas [15,16]. Metabolic risk factors, such as hypertension, obesity and type 2 diabetes mellitus, likely contribute to rising CCA rates globally; although they confer a relatively small relative risk increase, the high overall prevalence of such risk factors in Western countries suggests a significant contribution to case numbers overall [15]. In recent observational data from the ENSCCA registry, diabetes mellitus was seen less commonly in pCCA compared with the whole CCA cohort (15.6% vs. 22.5%, respectively), whereas primary sclerosing cholangitis was more common in pCCA compared with the whole cohort (8.8% vs. 4.5%, respectively) [17].

As previously mentioned, CCA generally presents at an advanced stage; the typical presentation of pCCA is with biliary obstruction without mass formation, due to the ‘periductal infiltrating’ morphology noted in the majority of cases [17]. The Bismuth–Corlette classification subdivides pCCA according to which biliary ducts are involved [18]. The associated difficulty in obtaining adequate tissue in pCCA is a barrier to diagnosis, but crucially, also prevents molecular profiling analysis, resulting in the under-representation of pCCA in clinical trials of systemic therapy (see Table 1) and an impaired ability to identify and act on molecular targets in the clinic, respectively [19]. Regarding the limitations of subdividing CCA anatomically, both hilar and small bile ducts and ductules are present in the liver parenchyma at the perihilar and intrahepatic levels. Both pCCA and iCCA, therefore, have multiple potential cells of origin, and anatomical pCCA and iCCA may arise from the same cell type; thus, they have similar molecular landscapes [20]. Anatomical classifications, therefore, do not map perfectly onto the cells of origin or molecular pathology, which is highly pertinent when considering the role of targeted therapies.

## 2. Molecular Characterisation of pCCA

In 2017, a large study of CCA (featuring 310 iCCAs, 128 pCCAs and 40 dCCAs) integrated whole-genome and epigenomic data to identify four distinct molecular “clusters”, and demonstrated that survival was better predicted by classification using those molecular subtypes than by the anatomical site of origin [25]. Clusters 3 and 4 were composed almost entirely of iCCA (92% vs. 8% pCCA; *p* < 0.001); CCA in Clusters 1 and 2 comprised a combination of anatomical subtypes, and conferred a worse prognosis (*p* < 0.001), including when fluke status was considered (*p* < 0.05). Cluster 1 was formed of 93% fluke-positive tumours, whereas Cluster 2 was 64% fluke-negative. The representation of 29 pCCA samples which were matched to a cluster was 38%, 48% and 14% for Clusters 1,2 and 4, respectively. Cluster 2 tumours featured higher rates of *TP53* mutations and human epidermal growth factor-2 (*HER2*) amplifications (*p* < 0.001 and *p* < 0.01, respectively) and increased HER2 expression (*p* < 0.05) [25].

A transcriptomic analysis of 189 cases of eCCA reported in 2020 proposed classifying eCCA into the following subtypes—immune, mesenchymal, metabolic and proliferation (11%, 47%, 19% and 23% of the cohort, respectively). Notably, 76% of the evaluated samples were pCCAs, and structural genetic alterations were not significantly different between pCCA and dCCA samples. Immune-type eCCAs were characterised by more intense lymphocytic infiltration, PD-1/PD-L1 overexpression and a corresponding improved response to immune checkpoint inhibition. Mesenchymal-type eCCAs demonstrated poorer survival and increased cell plasticity, secondary to epithelial–mesenchymal transition, with aberrant signalling via transforming growth factor beta and tumour necrosis factor alpha. Metabolic-type eCCAs demonstrated a hepatocyte-like phenotype with prominent derangement of bile acid metabolism; meanwhile, the proliferation subtype featured under-representation of pCCA, along with higher rates of MYC overexpression, *HER2* aberrations and activation of the RAS–RAF–mitogen-activated protein kinase kinase–extracellular-signal-regulated kinase (RAS–RAF–MEK–ERK) pathway [26].

**Figure 1:**
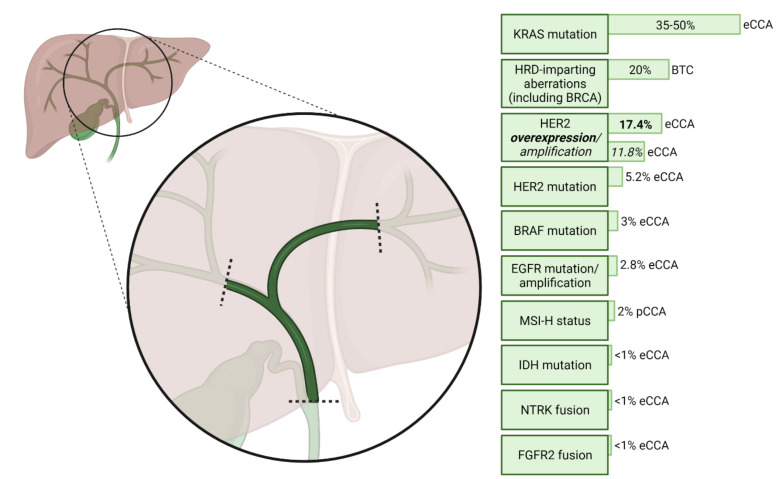
Frequency of potentially actionable molecular aberrations in perihilar cholangiocarcinoma. Anatomical groupings of patients sampled to determine each frequency are beside/below the corresponding frequency [25,26,27,28,29,30,31,32,33,34,35,36]. Rates of co-occurrence of the described molecular aberrations within patients are unknown. Created with BioRender.com. KRAS, Kirsten rat sarcoma virus. eCCA, extrahepatic cholangiocarcinoma. HRD, homologous repair deficiency. BRCA, breast cancer susceptibility gene. BTC, biliary tract cancer. HER2, human epidermal growth factor receptor 2. BRAF, v-Raf murine sarcoma viral oncogene homolog B. EGFR, epidermal growth factor receptor. MSI-H, microsatellite instability high. pCCA, perihilar cholangiocarcinoma. IDH, isocitrate dehydrogenase. NTRK, neurotrophic tropomyosin receptor kinases. FGFR2, fibroblast growth factor receptor 2.

Figure 1 summarises the frequency of potentially actionable molecular aberrations in pCCA. These molecular targets will now be discussed in further detail.

## 3. Potentially Actionable Molecular Aberrations in Perihilar Cholangiocarcinoma

### 3.1. Isocitrate Dehydrogenase-1

Isocitrate dehydrogenase (*IDH*) mutations are somatic gain-of-function mutations which typically occur early in tumour development to prevent normal cell differentiation and promote epigenetic dysregulation and carcinogenesis via accumulation of the oncometabolite 2-hydroxyglutarate [37,38]. Mutations in *IDH-1* are more common than those in *IDH-2*, and classically occur in fluke-negative, hepatitis-negative CCA [39,40]. In a 2019 systematic review, *IDH1* mutations were observed in 13% of 4214 iCCA cases, compared with 0.8% of 1123 eCCAs; no association with prognosis was identified [27]. Following impressive results in *IDH1*-mutant acute myeloid leukaemia [41] and a dose-escalation and expansion study within CCA—which included no patients with pCCA [42]—the phase 3 ClarIDHy trial demonstrated improved mPFS for ivosidenib versus placebo (2.7 vs. 1.4 months; HR 0.37, 95% CI 0.25–0.54; *p* < 0.0001) in patients with previously treated, *IDH1*-mutated CCA, and was subsequently FDA-approved in this setting [21,43]. The subsequently published mOS for ivosidenib was also superior to the placebo (10.3 vs. 7.5 months; HR 0.79, 95% CI 0.56–1.12; *p =* 0.093); patients who progressed on the placebo were subsequently offered ivosidenib, and as such a rank-preserving structural failure time model was employed, which adjusted the mOS in the placebo group to 5.1 months (HR 0.49, 95% CI 0.34–0.70; *p* < 0.0001) [44]. Only 2.2% of patients in this trial had pCCA, limiting the assessment of benefits according to anatomical subtype [21].

### 3.2. Fibroblast Growth Factor Receptor

The fibroblast growth factor receptor family of trans-membrane receptors has five members (FGFR 1–5). Fibroblast growth factor receptors 1–4 contain intracellular tyrosine kinase domains which, when activated, trigger signalling through multiple pathways including the RAS–RAF–MEK–ERK, Janus kinase/signal transducer and activator of transcription (JAK/STAT) and phosphatidylinositol-3-kinase/protein kinase B/mammalian target of rapamycin (PI3K/AKT/mTOR) pathways, which are heavily implicated in cell proliferation, differentiation and migration [45,46,47,48]. A sequencing study of 115 CCA cases found that aberrations of *FGFR2* are most common (6.1%, vs. 0.9% in *FGFR1*; 0% for *FGFR3–5*) with rearrangements/fusions (3.5%) more frequent than amplifications (2.6%) or mutations (0.9%) [49]. FGFR-targeted tyrosine kinase inhibitors (TKIs) have been developed, although patients with *FGFR2* fusions appear to be the only group who present responses to the majority of these [50,51,52,53,54,55]. Further sequencing studies have identified *FGFR2* fusions in approximately 14% of iCCA, and noted apparent mutual exclusivity with *IDH* mutations but identified no *FGFR1–3* aberrations in a combined 94 cases of eCCA [28,29]. Regarding the FGFR-TKIs in the most advanced stages of clinical development, phase 2/3 trials of futibatinib and derazantinib have specified iCCA as an inclusion criterion [56,57,58,59,60], whereas those of infigratinib and pemigatinib have permitted *FGFR2* fusion-positive eCCA (NCT03773302, NCT03656536). Among completed phase 2/3 trials of these drugs, only 1 patient with eCCA was included among a combined 190 enrolled patients with FGFR2 fusions [50,61]. Pemigatinib and infigratinib are FDA-approved for previously treated *FGFR2* fusion-positive CCA [62,63] on the basis of phase 2 trials which demonstrated a mPFS of 6.9–7.3 months and an overall response rate (ORR) of 23.1–35.5%, and the former demonstrating a mOS of 21.1 months [61,64,65,66]. In summary, there is little evidence that patients with pCCA have the required molecular alteration to derive benefit from demonstrably effective FGFR-targeted therapies.

### 3.3. The Epidermal Growth Factor Receptor Family

The four members of the epidermal growth factor receptor family (EGFR/HER1, HER2/3/4) are tyrosine kinases whose activation, by various possible ligands, causes dimerisation to produce either a homodimer or a heterodimer with another receptor of the same family. Heterodimers containing HER2 are particularly potent signal transducers [66]. Downstream effects of EGFR family member activation include activation of the RAS–RAF–MEK–ERK, JAK/STAT and PI3K/AKT/mTOR pathways [67]. Epidermal growth factor receptor expression, determined by IHC, has been observed in 19.2% of 130 eCCA cases, and confers significantly higher rates of lymph node metastasis, lymphatic vessel invasion and perineural invasion [68]. The overexpression of EGFR and HER2 has been identified as an independent marker of poor prognosis within BTC [69,70,71]. Somatic activating *EGFR* mutations occur in eCCA, but their frequency within pCCA is difficult to determine, because data on mutational frequency are rarely stratified by anatomical subtype [72]—somatic *EGFR* mutations were found in 9.5% of 137 resected BTCs in one study [73], and in 13.6% of 22 consecutive resected CCAs in another [74]. A next-generation sequencing study featuring 248 eCCAs revealed that 9.7% of patients harboured aberrations within the EGFR family, of which 53.6% had mutated *HER2* [30]. A further meta-analysis of 11 studies of eCCA found a mean HER2 overexpression rate (defined as moderate/strong IHC staining) of 17.4% (95% CI 3.4–31.4%) by IHC, with an HER2 amplification rate by in-situ hybridisation of 57.6% among tumours with moderate/strong expression by IHC [31].

Epidermal growth factor receptor-targeted therapies have demonstrated efficacy in other solid tumours including non-small cell lung cancer, and mutational status predicts response to such therapies [75]. Mutations of the K isoform of *RAS* (*KRAS*) serve as a strong negative predictor of anti-EGFR efficacy [76]. Conversely, the overexpression of HER2 in gastric and breast cancer is an established biomarker for efficacy of anti-HER2 treatment [77,78].

A randomised phase 3 study compared erlotinib, an EGFR TKI, with gemcitabine/oxaliplatin (GEMOX) chemotherapy as a first-line treatment in patients with metastatic BTC, without molecular selection. There was no significant difference in PFS, the primary endpoint, between GEMOX alone and alongside erlotinib (4.2 vs. 5.8 months, respectively; HR = 0.80, 95% CI 0.61–1.03; *p* = 0.087). The ORR was higher in the erlotinib arm (29.6% vs. 15.8%, *p* = 0.005), whereas mOS was 9.5 months in both arms. On subgroup analysis, erlotinib did appear to significantly prolong mPFS for patients with CCA (5.9 vs. 3.0 months; HR 0.73, 95% CI 0.53–1.00; *p* = 0.049); further stratification by anatomical subgroup was unfortunately not published [79].

The EGFR mAb cetuximab was assessed in a single-arm phase 2 study alongside GEMOX chemotherapy as a first-line treatment for 30 patients with advanced (unresectable or metastatic) BTC; the ORR was 63% (including 3 complete responses), and 9 patients underwent curative-intent resection following treatment [80]. However, further trials of EGFR mAbs have not shown a strong signal of activity, as displayed in Table 2. In the BINGO trial, EGFR overexpression (identified in 23% of 77 cases) and KRAS mutation (19% of 75 cases) were not found to be predictive of treatment response or outcome [81].

Previous small clinical trials evaluating lapatinib, a combined EGFR/HER2 TKI, in populations unselected for receptor expression status, failed to show efficacy in BTC [84,85]. However, small retrospective reviews and case reports of the ‘on-target’ use of HER2 inhibition, in patients with overexpressed or mutated HER2, have suggested clinical activity; this includes a 14-patient series demonstrating activity in GBC and a case report suggesting activity in eCCA [71,86]. The single-arm phase 2 SUMMIT basket trial evaluated the pan-HER irreversible TKI neratinib in 25 patients with previously treated BTC and somatic HER2 mutation. The ORR was 13% (95% CI 3–31%), mPFS was 2.8 months (95% CI 1.1–3.7) and mOS was 5.4 months (95% CI 3.7–11.7). Five patients with eCCA were included, but the results were not stratified by anatomical subtype [87]. A further phase 2 basket trial, the MyPathway study, has provided the largest prospective cohort of HER-2 positive (overexpression, amplification or both) patients with advanced BTC to date; 39 patients were treated with the HER2 mAbs pertuzumab and trastuzumab. All patients had previously been treated, with a median of two prior lines of therapy. The ORR was 23%, with a mPFS of 4.0 months (95% CI 1.8–5.7) and mOS of 10.9 months (95% CI 5.2–15.6). Among the seven patients with eCCA included within this cohort, the disease control rate (DCR) was 71%, with two partial responses, a mPFS of 6.8 months (95% CI 1.3–13.5) and a mOS of 8.0 months (2.0—not estimable) [24]. The authors favourably compared ORR, DCR and mOS with second-line FOLFOX, as reported in the ABC-06 trial. Although comparisons between trials are inherently problematic, this is encouraging, because the MyPathway trial enrolled patients with an Eastern Co-operative Oncology Group performance status (ECOG PS) of 0 to 2, its patients were more heavily pre-treated versus ABC-06, and also because HER2 positivity has historically conferred a worse prognosis among patients with BTC [12,70,86,88]. A single-arm phase 2b trial of zanidatamab, an HER2-bispecific antibody, planned for 100 patients with advanced HER2-amplified BTC, is currently recruiting (NCT04466891).

### 3.4. Microsatellite Instability and Immune Checkpoint Inhibition

A microsatellite instability-high (MSI-H) state arises from either germline mutations in components of the DNA mismatch repair (MMR) machinery in patients with Lynch syndrome, or somatic hypermethylation of the *MLH1* promoter, causing deficient MMR (dMMR) [89]. The rate of somatic mutations in MSI-H cancers is one to two orders of magnitude higher than microsatellite-stable cancers, with an increased generation of neoepitopes, an intense lymphocytic infiltrate, and superior prognosis, which may be capitalised on by PD-1/PD-L1 blockade [90]. An MSI-H status is rare in BTC—it has been observed in 1.9% of 106 patients with non-fluke-associated pCCA, 2.4% of 42 pCCAs in another German series, and in 13.2% of 38 patients with eCCA in a further Japanese series [32,91,92]. Notably, these studies all used resection specimens for analysis, which may represent a bias in selection, limiting generalisability to pCCA as a whole. Factors determined to be predictive of MSI-H include a younger age at diagnosis and atypical histomorphology [32,93].

Multiple case reports exist of patients with MSI-H CCA treated with the PD-1 antibody pembrolizumab with partial responses achieved, including a patient with eCCA who achieved a partial response lasting in excess of 13 months [94,95,96,97]. Some efficacy has been demonstrated by pembrolizumab in pre-treated PD-L1-expressing CCA/GBC with negative or unknown MSI status, with three studies showing ORRs of 9.8–13%, mPFS of 1.5–2.1 months and mOS of 4.3–6.9 months [94,98,99]. However, the 2019 phase 2 KEYNOTE-158 basket trial of pembrolizumab for previously treated MSI-H cancer included 22 patients with CCA, for whom the ORR was 40.9% (95% CI 20.7–63.6), with a mOS of 24.3 months (95% CI 6.5—not reached) [22], demonstrating the value of biomarkers in appropriately targeting therapy. Unfortunately, results were not stratified by CCA subtype. Pembrolizumab is FDA-approved as a second-line therapy for patients with MSI-H cancers which have progressed through prior therapy [100], and thus represents a promising treatment option in a small subset of patients with pCCA. The phase 3 TOPAZ-1 trial, published in abstract form following the 2022 American Society of Clinical Oncology Gastrointestinal Cancers Symposium, studied 685 patients with advanced BTC (not selected for markers of immunotherapeutic efficacy); the addition of the anti-PD-L1 mAb durvalumab, versus placebo, to first-line Cis/Gem chemotherapy improved overall survival (HR 0.80; 95% CI 0.64–0.89, *p* = 0.001; mOS 12.8 versus 11.5 months). Improvements in survival with the addition of durvalumab were most notable at later time points (2-year OS 24.9 vs. 10.4%), and the authors propose Cis/Gem plus durvalumab as a potential new standard-of-care regimen in the first line [11]. The impact on the pCCA sub-group may be available in due course. The phase 3 randomised KEYNOTE-966 (NCT04003636) study is ongoing, and evaluates pembrolizumab in the same setting.

### 3.5. Anti-Angiogenic Therapies

The induction of angiogenesis is crucial in sustaining neoplastic growth [101], and pCCA has been noted to exhibit a high degree of vascularisation, as measured by microvessel density (MVD). A high MVD is associated with higher rates of nodal spread and post-operative local recurrence, and confers poor prognosis in pCCA and other BTC subtypes [102,103,104]. Vascular endothelial growth factor (VEGF) is critical in initiating angiogenesis, and has been found by immunohistochemistry (IHC) to be expressed in 59.2% of 130 eCCA tumours in a large series [68]; a further study of 111 eCCA cases suggested expression by IHC in 69% of dCCA tumours compared with only 25% of pCCA (*p* < 0.0001) [105]. Trials of VEGF inhibition in BTC have included VEGF monoclonal antibodies (mAbs) such as bevacizumab, or multi-receptor TKIs, and will now be discussed.

Bevacizumab inhibits the ligand VEGF-A, and its limited efficacy as a monotherapy has been attributed to activation of alternative pathways which overcome the anti-angiogenic activity of VEGF inhibition. Tyrosine kinase inhibitors, however, block multiple signalling pathways, some of which are directly implicated in tumour growth rather than purely angiogenesis [106,107,108,109]; inhibiting multiple targets thereby offers the potential for increased efficacy, at the cost of higher toxicity [110]. The theory of tumour vascular normalisation, whereby anti-angiogenic therapies transiently normalise tumour blood vessels to improve the delivery of oxygen and medications [111], advocates for combinations with other treatments to which anti-angiogenic therapy might sensitise the tumour. However, trials of combinations with both cytotoxic chemotherapy and other targeted therapies, in addition to those of TKI monotherapy, have thus far failed to demonstrate superiority to standard-of-care treatments, as demonstrated in Table 3. This includes most recently the randomised phase 2 trial of first-line ramucirumab or merestinib in addition to Cis/Gem, which did not prolong PFS in a molecularly unselected cohort [112]. The phase 2 LEAP-005 trial (NCT03797326) is ongoing, and combines TKI therapy with immune checkpoint inhibition, an area of significant investigational interest [113].

### 3.6. The RAS–RAF–MEK–ERK Pathway

The RAS–RAF–MEK–ERK pathway constitutes a major signalling component of the mitogen-activated protein kinase (MAPK) cascade, which transduces signals from the intracellular aspect of the cell membrane to the nucleus. Downstream nuclear effectors modulate processes such as cell growth, differentiation and survival. Accordingly, the RAS–RAF–MEK–ERK pathway is commonly dysregulated by activating mutations during oncogenesis [129].

Mutations of *KRAS* are represented in different proportions across all anatomical subtypes of CCA and are associated with a worse prognosis [25,28], although they are particularly prevalent in eCCA with a frequency of 35–50% [26,33,34], making them among the most frequent known mutations in eCCA. Decades of effort towards developing RAS inhibitors have been without success; therefore, the perception of RAS as ‘undruggable’ had become established [130]. However, in 2021, following evidence of durable clinical benefit in a Phase 2 trial, the FDA granted accelerated approval to sotorasib, a mutation-specific RAS GTPase inhibitor, in *KRAS^G12C^*-mutated non-small cell lung cancer [131,132]. Adagrasib is a further KRAS^G12C^ inhibitor which was evaluated in the phase 2 KRYSTAL-1 trial; recently published preliminary results from 17 patients with non-pancreatic GI cancers, including 8 patients with BTC, suggested a DCR of 100% with an ORR of 35%, although the representation of pCCA in this cohort is unclear [133]. The *KRAS^G12C^* mutation has been identified in 15% of 20 eCCA tumours in one study, but in only 1.5% of a larger study of 130 eCCA cases [26,34]; where identified, however, such patients may be considered for future basket trials.

Given the historical absence of effective KRAS inhibitors, efforts to prevent oncogenic signalling via the RAS–RAF–MEK–ERK pathway have focused on inhibition of the downstream targets of RAS. Activating mutations of the B isoform of *RAF* (*BRAF*) occur in CCA, and are less common in eCCA versus iCCA—5% of 412 iCCAs and 3% of 57 eCCA in one study [28]. A further study of 203 patients from China with eCCA reported a proportion of 8.9% [134]. A recent analysis of 54 patients with iCCA with the *BRAF^V600E^* point mutation reported the mutation to be associated with a more advanced TNM stage, resistance to chemotherapy and worse OS [135,136]. Earlier trials using BRAF inhibitor monotherapy showed limited activity [137]; therefore, more recently clinical trials have focused on BRAF–MEK inhibitor combination therapy, following superior outcomes in other malignancies such as metastatic melanoma for patients with *BRAF^V600E^* mutations [138,139]. To this end, the ROAR basket trial evaluated dabrafenib and trametinib in 43 patients with previously treated, *BRAF^V600E^*-mutated BTC; 1 patient with pCCA was included. For those 43 patients, the independently assessed ORR was 47% (95% CI 31–62), whereas the median response duration and mPFS were both 9 months (95% CI 6–14, and 5–10, respectively), and the mOS was 14 months (95% CI 10–33) [23]. Although *BRAF^V600E^* mutations are rare in BTC, the benefit for those found to have the mutation makes routine testing worth considering.

The MEK inhibitor selumetinib showed promising clinical activity in a phase 2 trial of 28 patients with advanced BTC—an ORR of 12%, stable disease in 68% of patients, and mOS of 9.8 months [140]. These findings were supported by the subsequent phase 1b ABC-04 study, which assessed selumetinib as first-line therapy in combination with Cis/Gem; of eight patients enrolled, partial responses were seen in three and stable disease in the remaining five, with a mPFS of 6.4 months [141]. On the basis of these data, the randomised phase 2 BIL-MEK study of 57 patients assessed the addition of selumetinib, either continuous or sequentially dosed, to Cis/Gem as first-line therapy, without molecular selection. The addition of selumetinib, however, failed to improve tumour response at 10 weeks, the study’s primary endpoint, with either dosing schedule [142].

The first-in-class ERK 1/2 inhibitor ulixertinib demonstrated early evidence of clinical activity in a phase 1 dose-escalation and expansion study of MAPK-mutated advanced malignancies. The ORR was 14% of 81 evaluable patients in the dose-escalation cohort, and notable responses were seen in non-*BRAF^V600^*-mutated tumours [143]. A phase 2 trial of previously treated MEK/BRAF-mutated advanced malignancies is recruiting to further evaluate ulixertinib (NCT04488003).

### 3.7. Other Targeted Therapies

Poly ADP-ribose polymerase (PARP) inhibitors are utilised in cancers with homologous repair deficiency (HRD), to deliver the second of two ‘hits’ to tumour cells with pre-existing DNA damage repair (DDR) defects; without functional PARP enzymes, double-stranded DNA breaks occur, and cell death follows [144]. Various DDR gene alterations may produce HRD—notably including *IDH1* and *IDH2* mutations [145]—and are identified in up to 20% of BTC, with a higher rate observed in eCCA [35]. Phase 2 clinical trials are ongoing to assess PARP inhibitors in BTC, as monotherapy (NCT04042831, NCT03207347, NCT03212274) and combined with PD-1/PD-L1 inhibitors (NCT04895046, NCT04306367) or temozolomide (NCT04796454).

Activated mTOR has been identified by IHC in 64% of 88 BTCs in one series, including 66% of 15 eCCAs, and is further identified as a negative prognostic marker [146]; the upregulation of downstream effectors of mTOR signalling is also seen in BTC [147,148]. The mTOR inhibitor everolimus has demonstrated limited efficacy, with a mPFS of 3.2 months (95% CI 1.8–4.0) and mOS of 7.7 months (95% CI 5.5–13.2) in a Phase 2 trial of patients with pre-treated advanced BTC [149]. In a further phase 2 trial, as a first-line monotherapy, the mPFS with everolimus was 5.5 months (95% CI 2.1–10.0) and the mOS was 9.5 months (95% CI 5.5–16.6) [150]. In a recent phase 2 trial of advanced BTC, the pan-PI3K inhibitor copanlisib was tested as first-line treatment in combination with Cis/Gem and demonstrated a mPFS of 6.2 months (95% CI 2.9–10.1) and a mOS of 13.7 months (95% CI 6.8–18.0), failing to meet the primary endpoint of improved PFS at 6 months [151].

The neurotrophic tropomyosin receptor kinases are a family of tyrosine kinases with three members (NTRK-1/2/3) which may undergo fusions causing their constitutive activation, contributing to oncogenesis in multiple cancers [152,153]. Such fusions have been identified in CCA [154,155], and 2 patients with previously treated NRTK-positive eCCA were studied in a 55-patient phase 2 trial of larotrectinib, an NRTK TKI. An ORR of 75% (95% CI 61–85) was seen, with 55% of patients remaining progression-free at 1 year of follow-up [156]. These encouraging results were tempered by a more recent sequencing study of 149 BTCs, which identified only one NTRK fusion (a pCCA), for an overall frequency of 0.67% [36]. Two basket trials of NTRK TKIs in patients with previously treated BTC are ongoing (NCT02568267 and NCT02576431).

## 4. Conclusions

In recent years, a multitude of potential avenues for targeted therapies within CCA has been explored (as summarised in Figure 1) and most, usually in unselected patient populations, have failed to translate a signal of efficacy into larger clinical trials. The recently published TOPAZ-1 study [11] is an exception in this regard, where the addition of a targeted therapy has produced a signal of efficacy despite a lack of molecular selection; the direct applicability of these results to pCCA is awaited. A few treatments have previously demonstrated impressive results in molecularly selected CCA patients with rare molecular aberrations; however, regarding pCCA, such trials have either included only a low proportion of patients with pCCA, or have not specified how many enrolled patients actually have pCCA. Therefore, the importance of reporting anatomical subtype in trials featuring patients with CCA cannot be overstated. Targeted therapies relevant to pCCA include pembrolizumab in patients with MSI-H disease, ivosidenib for IDH-1-mutant disease, and the pertuzumab/trastuzumab combination for HER-2-positive disease.

As our understanding of the molecular landscape of CCA has grown, both the importance and limitations of anatomical subtyping have become increasingly clear. Although certain genomic and epigenomic profiling studies have characterised molecular subtypes with greater prognostic power than anatomical subtyping, the ubiquity of imaging for diagnosis and difficulties obtaining tissue for molecular profiling, particularly in pCCA, dictate that anatomical location remains the primary means of subdividing CCA. Although liquid biopsy techniques may alter this paradigm in the future, they are some way from being utilised in routine clinical practice; future classifications of CCA should aim to account for radiological, histological and molecular features of the disease [2]. In the future, the importance of molecular profiling in CCA is set to further increase; profiling should be considered early in a patient’s disease course because results typically take weeks (representing a barrier to first-line adoption of targeted therapies in molecularly selected patients).

The present review illustrates both the complexity and diversity of molecular aberrations in pCCA. Progress is being made towards the goal of personalised medicine, with the availability of a range of effective agents targeted against the molecular drivers of patients’ cancers, but presently, only a small proportion of patients can benefit. Continuing to expand our understanding of the genomic and epigenomic landscape of CCA is essential in developing further targeted therapies, understanding past failures, and in maximising the utility of existing treatments by identifying accurate biomarkers or opportunities for treatment combinations.

## Figures and Tables

**Table 1:** Representation of subtypes of biliary tract cancer in selected clinical trials of chemotherapy and targeted therapies.

Trial; Number of Patients Enrolled	iCCA	pCCA	dCCA	GBC	AVC
ABC-02 [9]; *n* = 410	241 (59% CCA)			149 (36%)	20 (5%)
BT22 [10]; *n* = 83	28 (34%)	19 (23% eCCA)		32 (39%)	4 (5%)
ABC-06 [12]; *n* = 162	72 (44%)	45 (28% eCCA)		34 (21%)	11 (7%)
NIFTY [13]; *n* = 174	74 (43%)	47 (27% eCCA)		53 (30%)	0
ClarIDHy [21]; *n* = 185	169 (91%)	4 (2%)	2 (1%)	0	0
KEYNOTE-158 [22] (CCA patients only); *n* = 22	22 (100%; CCA subtype not reported)			0	0
ROAR [23]; *n* = 43	39 (91%)	1 (2%)	0	1 (2%)	0
MyPathway [24] (BTC patients only); *n* = 39	7 (18%)	7 (18% eCCA)		16 (41%)	5 (13%)

Note that a small proportion of enrolled patients had an ‘unknown’ anatomical subtype; hence, the sum of all subtyped patients may not equal the total enrolled for a given study. iCCA/pCCA/dCCA, intrahepatic/perihilar/distal cholangiocarcinoma. GBC, gallbladder cancer. AVC, ampulla of Vater carcinoma. BTC, biliary tract cancer.

**Table 2:** Selected trials of monoclonal antibodies against the epidermal growth factor receptor in advanced biliary tract cancer.

Trial; Year of Publication	KRAS Selection	Treatment Arm(s); Number of Patients	ORR	mPFS (95% CI)	mOS (95% CI)
Gruenberger et al., 2010 [80]	None	Gem/Ox + cetuximab (*n* = 30)	63% (including 10% CR)	-	-
Malka et al. (BINGO trial), 2014 [81]	None	Gem/Ox (*n* = 74)	23% (including 3% CR)	5.5 (3.7–6.6)	12.4 (8.6–16.0)
		Gem/Ox + cetuximab (*n* = 76)	24% (including 1% CR)	6.1 (5.1–7.6)	11.0 (9.1–13.7)
Leone et al. (Vecti-BIL trial), 2016 [82]	Wild-type only	Gem/Ox (*n* = 44)	18% (including 2% CR)	4.4 (2.6–6.2)	10.2 (6.4–13.9)
		Gem/Ox + panitumumab (*n* = 45)	27% (including 2% CR)	5.3 (3.3–7.2); *p* = 0.27	9.9 (5.4–14.3)
Vogel et al. (PICCA trial), 2018 [83]	Wild-type only	Cis/Gem (*n* = 28)	39% (% CR unknown)	-	20.1
		Cis/Gem + panitumumab (*n* = 62)	45% (% CR unknown)	-	12.8

All trials shown are phase 2. All figures for mPFS and mOS are in months; ‘advanced’ biliary tract cancer refers to unresectable or metastatic disease. KRAS, Kirsten rat sarcoma virus. mPFS, median progression-free survival. ORR, objective response rate. mOS, median overall survival. Gem, gemcitabine. Ox, oxaliplatin. CR, complete response. Cis, cisplatin.

**Table 3:** Selected clinical trials of vascular endothelial growth factor inhibition in advanced biliary tract cancer.

Trial; Year of Publication	Setting	Treatment Arm(s); Number of Patients	ORR	mPFS (95% CI)	mOS (95% CI)
**VEGF inhibition as monotherapy**					
Bengala et al., 2010 [114]	1st- or 2nd-line	Sorafenib (*n* = 46)	2%	2.3	4.4
Yi et al., 2012 [105]	2nd-line	Sunitinib (*n* = 56)	8.9%	1.7 (1.0–2.4)	4.8 (3.8–4.8)
El-Khoueiry et al. (SWOG 0514 trial), 2012 [115]	1st-line	Sorafenib (*n* = 31)	0%	3.0 (2.0–4.0)	9.0 (4.0–12.0)
Sun et al., 2019 [116]	2nd-line	Regorafenib (*n* = 43)	11%	15.6 wk	31.8 wk
Demols et al. (REACHIN trial), 2020 [117]	2nd-line	Regorafenib (*n* = 33)	0%	3.0 (2.3–4.9)	5.3 (2.7–10.5)
		Placebo (*n* = 33)	0%	1.5 (1.2–2.0)	5.1 (3.0–6.4)
Ueno et al., 2020 [118]	2nd-line	Lenvatinib (*n* = 26)	11.5%	1.6 (1.4–3.2)	7.35 (4.5–11.3)
**Chemotherapy plus VEGF inhibition**					
Zhu et al., 2010 [119]	1st-line	Gem/Ox plus bevacizumab (*n* = 35)	40%	7 (5.3–10.3)	12.7 (7.3–18.1)
Iyer et al., 2018 [120]	1st-line	Gem/Cap plus bevacizumab (*n* = 50)	24%	8.1 (5.3–9.9)	10.2 (7.5–13.7)
Lee et al., 2013 [121]	1st-line	Cis/Gem plus sorafenib (*n* = 39)	12%	6.5 (3.5–8.3)	14.4 (11.6–19.2)
Moehler et al., 2014 [122]	1st-line	Gem plus sorafenib (*n* = 49)	14%	3.0	8.4
		Gem plus placebo (*n* = 48)	10%	4.9	11.2
Santoro et al. (VanGogh trial), 2015 [123]	1st-line	Vandetanib (*n* = 56)	3.6%	15 wk (10.3–22.1)	32.6 wk (27.1–52)
		Vandetanib plus Gem (*n* = 57)	19.3%	16.3 wk (13–27.6)	40.6 wk (30.4–51.3)
		Placebo plus Gem (*n* = 52)	13.5%	21.1 wk (10.1–32.1)	43.9 wk (36.3–74.7)
Jensen et al., 2015 [124]	1st-line	Panitumumab + Gem/Ox/Cap (*n* = 44)	46%	6.1 (5.8–8.1)	9.5 (8.3–13.3)
		Gem/Ox/Cap + bevacizumab (*n* = 44)	18%	8.2 (5.3–10.6)	12.3 (8.8–13.3)
Valle et al. (ABC-03 trial), 2015 [125]	1st-line	Cis/Gem plus cediranib (*n* = 62)	44% (including 3% CR)	8 (6.5–9.3)	14.1 (10.2–16.4)
		Cis/Gem plus placebo (*n* = 62)	19%	7.4 (5.7–8.5)	11.9 (9.2–14.3)
Valle et al., 2021 [112]	1st-line	Cis/Gem plus ramucirumab (*n* = 106)	31.1%	6.5 (80% CI 5.7–7.1)	10.5 (80% CI 8.5–11.8)
		Cis/Gem plus merestinib (*n* = 102)	19.6%	7.9 (80% CI 6.2–7.1)	14.0 (80% CI 12.0–16.4)
		Cis/Gem plus placebo (*n* = 101)	32.7% (including 2% CR)	6.6 (80% CI 5.6–6.8)	13.0 (80% CI 11.4–15.3)
**Targeted therapy combinations including VEGF inhibition**					
Lubner et al., 2010 [126]	1st-line	Bevacizumab plus erlotinib (*n* = 49)	12%	4.4 (3–7.8)	9.9 (7.2–13.6)
El-Khouiery et al. (SWOG S0941 trial), 2014 [127]	1st-line	Sorafenib plus erlotinib (*n* = 34)	6%	2 (2–3)	6 (3–8)
Lin et al., 2020 [128]	2nd-line	Lenvatinib plus pembrolizumab (*n* = 32)	25%	4.9 (4.7–5.2)	11 (9.6–12.3)

All figures for mPFS and mOS are in months unless otherwise shown; ‘advanced’ biliary tract cancer refers to unresectable or metastatic disease. ORR, objective response rate. mPFS, median progression-free survival. mOS, median overall survival. VEGF, vascular endothelial growth factor. Gem, gemcitabine. Cap, capecitabine. Ox, oxaliplatin. Cis, cisplatin. CR, complete response. Wk, week(s).

## References

[1] Valle J.W., Kelley R.K., Nervi B., Oh D.-Y., Zhu A.X. (2021). Biliary tract cancer. Lancet.

[2] Banales J.M., Marin J.J.G., Lamarca A., Rodrigues P.M., Khan S.A., Roberts L.R., Cardinale V., Carpino G., Andersen J.B., Braconi C. (2020). Cholangiocarcinoma 2020: The next horizon in mechanisms and management. Nat. Rev. Gastroenterol. Hepatol..

[3] Brunt E., Aishima S., Clavien P.-A., Fowler K., Goodman Z., Gores G., Gouw A., Kagen A., Klimstra D., Komuta M. (2018). cHCC-CCA: Consensus terminology for primary liver carcinomas with both hepatocytic and cholangiocytic differentation. Hepatology.

[4] Rizvi S., Gores G.J. (2013). Pathogenesis, Diagnosis, and Management of Cholangiocarcinoma. Gastroenterology.

[5] Groot Koerkamp B., Wiggers J.K., Allen P.J., Besselink M.G., Blumgart L.H., Busch O.R., Coelen R., D’Angelica M.I., DeMatteo R.P., Gouma D.J. (2015). Recurrence Rate and Pattern of Perihilar Cholangiocarcinoma after Curative Intent Resection. J. Am. Coll. Surg..

[6] Komaya K., Ebata T., Shirai K., Ohira S., Morofuji N., Akutagawa A., Yamaguchi R., Nagino M., Aoba T., Kaneoka Y. (2017). Recurrence after resection with curative intent for distal cholangiocarcinoma. Br. J. Surg..

[7] Hyder O., Hatzaras I., Sotiropoulos G.C., Paul A., Alexandrescu S., Marques H.P., Pulitano C., Barroso E., Clary B.M., Aldrighetti L. (2013). Recurrence after operative management of intrahepatic cholangiocarcinoma. Surgery.

[8] Primrose J.N., Neoptolemos J., Palmer D.H., Malik H.Z., Prasad R., Mirza D., Anthony A., Corrie P., Falk S., Finch-Jones M. (2019). Capecitabine compared with observation in resected biliary tract cancer (BILCAP): A randomised, controlled, multicentre, phase 3 study. Lancet Oncol..

[9] Valle J., Wasan H., Palmer D.H., Cunningham D., Anthoney A., Maraveyas A., Madhusudan S., Iveson T., Hughes S., Pereira S.P. (2010). Cisplatin plus Gemcitabine versus Gemcitabine for Biliary Tract Cancer. N. Engl. J. Med..

[10] Okusaka T., Nakachi K., Fukutomi A., Mizuno N., Ohkawa S., Funakoshi A., Nagino M., Kondo S., Nagaoka S., Funai J. (2010). Gemcitabine alone or in combination with cisplatin in patients with biliary tract cancer: A comparative multicentre study in Japan. Br. J. Cancer.

[11] Oh D.-Y., He A.R., Qin S., Chen L.-T., Okusaka T., Vogel A., Kim J.W., Suksombooncharoen T., Lee M.A., Kitano M. (2022). A phase 3 randomized, double-blind, placebo-controlled study of durvalumab in combination with gemcitabine plus cisplatin (GemCis) in patients (pts) with advanced biliary tract cancer (BTC): TOPAZ-1. J. Clin. Oncol..

[12] Lamarca A., Palmer D.H., Wasan H.S., Ross P.J., Ma Y.T., Arora A., Falk S., Gillmore R., Wadsley J., Patel K. (2021). Second-line FOLFOX chemotherapy versus active symptom control for advanced biliary tract cancer (ABC-06): A phase 3, open-label, randomised, controlled trial. Lancet Oncol..

[13] Yoo C., Kim K.-P., Jeong J.H., Kim I., Kang M.J., Cheon J., Kang B.W., Ryu H., Lee J.S., Kim K.W. (2021). Liposomal irinotecan plus fluorouracil and leucovorin versus fluorouracil and leucovorin for metastatic biliary tract cancer after progression on gemcitabine plus cisplatin (NIFTY): A multicentre, open-label, randomised, phase 2b study. Lancet Oncol..

[14] ICD-11 for Mortality and Morbidity Statistics. https://icd.who.int/browse11/l-m/en.

[15] Clements O., Eliahoo J., Kim J.U., Taylor-Robinson S.D., Khan S.A. (2019). Risk factors for intrahepatic and extrahepatic cholangiocarcinoma: A systematic review and meta-analysis. J. Hepatol..

[16] Prueksapanich P., Piyachaturawat P., Aumpansub P., Ridtitid W., Chaiteerakij R., Rerknimitr R. (2018). Liver Fluke-Associated Biliary Tract Cancer. Gut Liver.

[17] Izquierdo-Sanchez L., Lamarca A., La Casta A., Buettner S., Utpatel K., Klümpen H.-J., Adeva J., Vogel A., Lleo A., Fabris L. (2021). Cholangiocarcinoma landscape in Europe: Diagnostic, prognostic and therapeutic insights from the ENSCCA Registry. J. Hepatol..

[18] Bismuth H., Nakache R., Diamond T. (1992). Management Strategies in Resection for Hilar Cholangiocarcinoma. Ann. Surg..

[19] Franssen S., de Jong D.M., van Driel L.M.J.W., Koerkamp B.G., Tabibian J.H. (2021). Challenges in Diagnosing Cholangiocarcinoma: Pulling Together Biochemical, Radiological, and Cytopathological Data. Diagnosis and Management of Cholangiocarcinoma.

[20] Hoadley K.A., Yau C., Hinoue T., Wolf D.M., Lazar A.J., Drill E., Shen R., Taylor A.M., Cherniack A.D., Thorsson V. (2018). Cell-of-Origin Patterns Dominate the Molecular Classification of 10,000 Tumors from 33 Types of Cancer. Cell.

[21] Abou-Alfa G.K., Macarulla T., Javle M.M., Kelley R.K., Lubner S.J., Adeva J., Cleary J.M., Catenacci D.V., Borad M.J., Bridgewater J. (2020). Ivosidenib in IDH1-mutant, chemotherapy-refractory cholangiocarcinoma (ClarIDHy): A multicentre, randomised, double-blind, placebo-controlled, phase 3 study. Lancet Oncol..

[22] Marabelle A., Le D.T., Ascierto P.A., Di Giacomo A.M., De Jesus-Acosta A., Delord J.-P., Geva R., Gottfried M., Penel N., Hansen A.R. (2020). Efficacy of Pembrolizumab in Patients with Noncolorectal High Microsatellite Instability/Mismatch Repair–Deficient Cancer: Results From the Phase II KEYNOTE-158 Study. J. Clin. Oncol. Off. J. Am. Soc. Clin. Oncol..

[23] Subbiah V., Lassen U., Élez E., Italiano A., Curigliano G., Javle M., de Braud F., Prager G.W., Greil R., Stein A. (2020). Dabrafenib plus trametinib in patients with BRAFV600E-mutated biliary tract cancer (ROAR): A phase 2, open-label, single-arm, multicentre basket trial. Lancet Oncol..

[24] Javle M., Borad M.J., Azad N.S., Kurzrock R., Abou-Alfa G.K., George B., Hainsworth J., Meric-Bernstam F., Swanton C., Sweeney C.J. (2021). Pertuzumab and trastuzumab for HER2-positive, metastatic biliary tract cancer (MyPathway): A multicentre, open-label, phase 2a, multiple basket study. Lancet Oncol..

[25] Jusakul A., Cutcutache I., Yong C.H., Lim J.Q., Ni Huang M., Padmanabhan N., Nellore V., Kongpetch S., Ng A.W.T., Ng L.M. (2017). Whole-Genome and Epigenomic Landscapes of Etiologically Distinct Subtypes of Cholangiocarcinoma. Cancer Discov..

[26] Montal R., Sia D., Montironi C., Leow W.Q., Esteban-Fabró R., Pinyol R., Torres-Martin M., Bassaganyas L., Moeini A., Peix J. (2020). Molecular classification and therapeutic targets in extrahepatic cholangiocarcinoma. J. Hepatol..

[27] Boscoe A.N., Rolland C., Kelley R.K. (2019). Frequency and prognostic significance of isocitrate dehydrogenase 1 mutations in cholangiocarcinoma: A systematic literature review. J. Gastrointest. Oncol..

[28] Javle M., Bekaii-Saab T., Jain A., Wang Y., Kelley R.K., Wang K., Kang H.C., Catenacci D., Ali S., Krishnan S. (2016). Biliary cancer: Utility of next-generation sequencing for clinical management. Cancer.

[29] Lowery M., Ptashkin R.N., Jordan E.J., Berger M.F., Zehir A., Capanu M., Kemeny N.E., O’Reilly E.M., El-Dika I., Jarnagin W.R. (2018). Comprehensive Molecular Profiling of Intrahepatic and Extrahepatic Cholangiocarcinomas: Potential Targets for Intervention. Clin. Cancer Res. Off. J. Am. Assoc. Cancer Res..

[30] Jacobi O., Ross J.S., Goshen-Lago T., Haddad R., Moore A., Sulkes A., Brenner B., Ben-Aharon I. (2020). ERBB2 Pathway in Biliary Tract Carcinoma: Clinical Implications of a Targetable Pathway. Oncol. Res. Treat..

[31] Galdy S., Lamarca A., Mcnamara M., Hubner R.A., Cella C.A., Fazio N., Valle J.W. (2016). HER2/HER3 pathway in biliary tract malignancies; systematic review and meta-analysis: A potential therapeutic target?. Cancer Metastasis Rev..

[32] Goeppert B., Roessler S., Renner M., Singer S., Mehrabi A., Vogel M.N., Pathil A., Czink E., Köhler B., Springfeld C. (2018). Mismatch repair deficiency is a rare but putative therapeutically relevant finding in non-liver fluke associated cholangiocarcinoma. Br. J. Cancer.

[33] Simbolo M., Fassan M., Ruzzenente A., Mafficini A., Wood L.D., Corbo V., Melisi D., Malleo G., Vicentini C., Malpeli G. (2014). Multigene mutational profiling of cholangiocarcinomas identifies actionable molecular subgroups. Oncotarget.

[34] Churi C.R., Shroff R., Wang Y., Rashid A., Kang H., Weatherly J., Zuo M., Zinner R., Hong D., Meric-Bernstam F. (2014). Mutation Profiling in Cholangiocarcinoma: Prognostic and Therapeutic Implications. PLoS ONE.

[35] Ahn D.H., Bekaii-Saab T. (2020). Biliary tract cancer and genomic alterations in homologous recombinant deficiency: Exploiting synthetic lethality with PARP inhibitors. Chin. Clin. Oncol..

[36] Demols A., Perez-Casanova L., Rocq L., Charry M., De Nève N., Verrellen A., Ramadhan A., Van Campenhout C., De Clercq S., Maris C. (2020). O-4 NTRK gene fusions in bilio-pancreatic cancers. Ann. Oncol..

[37] Mondesir J., Willekens C., Touat M., de Botton S. (2016). IDH1 and IDH2 mutations as novel therapeutic targets: Current perspectives. J. Blood Med..

[38] Borger D.R., Goyal L., Yau T.C.C., Poon R.T., Ancukiewicz M., Deshpande V., Christiani D.C., Liebman H.M., Yang H., Kim H. (2014). Circulating Oncometabolite 2-Hydroxyglutarate Is a Potential Surrogate Biomarker in Patients with Isocitrate Dehydrogenase-Mutant Intrahepatic Cholangiocarcinoma. Clin. Cancer Res. Off. J. Am. Assoc. Cancer Res..

[39] Chan-On W., Nairismägi M.-L., Ong C.K., Lim W.K., Dima S., Pairojkul C., Lim K.H., McPherson J.R., Cutcutache I., Heng H.L. (2013). Exome sequencing identifies distinct mutational patterns in liver fluke–related and non-infection-related bile duct cancers. Nat. Genet..

[40] Fujimoto A., Furuta M., Shiraishi Y., Gotoh K., Kawakami Y., Arihiro K., Nakamura T., Ueno M., Ariizumi S.-I., Nguyen H.H. (2015). Whole-genome mutational landscape of liver cancers displaying biliary phenotype reveals hepatitis impact and molecular diversity. Nat. Commun..

[41] Dinardo C.D., Stein E.M., DE Botton S., Roboz G.J., Altman J.K., Mims A.S., Swords R., Collins R.H., Mannis G.N., Pollyea D.A. (2018). Durable Remissions with Ivosidenib inIDH1-Mutated Relapsed or Refractory AML. N. Engl. J. Med..

[42] Lowery M.A., Burris H.A., Janku F., Shroff R.T., Cleary J.M., Azad N.S., Goyal L., Maher E.A., Gore L., Hollebecque A. (2019). Safety and activity of ivosidenib in patients with idh1-mutant advanced cholangiocarcinoma: A phase 1 study. Lancet Gastroenterol. Hepatol..

[43] Adeva J. (2021). Current development and future perspective of IDH1 inhibitors in cholangiocarcinoma. Liver Cancer Int..

[44] Zhu A.X., Macarulla T., Javle M.M., Kelley R.K., Lubner S.J., Adeva J., Cleary J.M., Catenacci D.V., Borad M.J., Bridgewater J.A. (2021). Final results from ClarIDHy, a global, phase III, randomized, double-blind study of ivosidenib (IVO) versus placebo (PBO) in patients (pts) with previously treated cholangiocarcinoma (CCA) and an isocitrate dehydrogenase 1 (IDH1) mutation. J. Clin. Oncol..

[45] Presta M., Chiodelli P., Giacomini A., Rusnati M., Ronca R. (2017). Fibroblast growth factors (FGFs) in cancer: FGF traps as a new therapeutic approach. Pharmacol. Ther..

[46] Alzahrani A.S. (2019). PI3K/Akt/mTOR inhibitors in cancer: At the bench and bedside. Semin. Cancer Biol..

[47] Thomas S.J., Snowden J.A., Zeidler M.P., Danson S.J. (2015). The role of JAK/STAT signalling in the pathogenesis, prognosis and treatment of solid tumours. Br. J. Cancer.

[48] Roberts P.J., Der C.J. (2007). Targeting the Raf-MEK-ERK mitogen-activated protein kinase cascade for the treatment of cancer. Oncogene.

[49] Helsten T., Elkin S., Arthur E., Tomson B.N., Carter J., Kurzrock R. (2015). The FGFR Landscape in Cancer: Analysis of 4,853 Tumors by Next-Generation Sequencing. Clin. Cancer Res. Off. J. Am. Assoc. Cancer Res..

[50] Javle M., Lowery M., Shroff R.T., Weiss K.H., Springfeld C., Borad M.J., Ramanathan R.K., Goyal L., Sadeghi S., Macarulla T. (2018). Phase II Study of BGJ398 in Patients with FGFR-Altered Advanced Cholangiocarcinoma. J. Clin. Oncol. Off. J. Am. Soc. Clin. Oncol..

[51] Mazzaferro V., El-Rayes B.F., Cotsoglou C., Harris W.P., Damjanov N., Masi G., Rimassa L., Personeni N., Braiteh F.S., Zagonel V. (2017). ARQ 087, an oral pan-fibroblast growth factor receptor (FGFR) inhibitor, in patients (pts) with advanced intrahepatic cholangiocarcinoma (iCCA) with FGFR2 genetic aberrations. J. Clin. Oncol..

[52] Abou-Alfa G.K., Sahai V., Hollebecque A., Vaccaro G., Melisi D., Al-Rajabi R., Paulson A.S., Borad M.J., Gallinson D., Murphy A.G. (2020). Pemigatinib for previously treated, locally advanced or metastatic cholangiocarcinoma: A multicentre, open-label, phase 2 study. Lancet Oncol..

[53] Cleary J.M., Voss M.H., Meric-Bernstam F., Hierro C., Heist R.S., Ishii N., Kirpicheva Y., Nicolas-Metral V., Pokorska-Bocci A., Vaslin A. (2018). Safety and efficacy of the selective FGFR inhibitor debio 1347 in phase I study patients with FGFR genomically activated advanced biliary tract cancer (BTC). J. Clin. Oncol..

[54] Bahleda R., Italiano A., Hierro C., Mita A., Cervantes A., Chan N., Awad M., Calvo E., Moreno V., Govindan R. (2019). Multicenter Phase I Study of Erdafitinib (JNJ-42756493), Oral Pan-Fibroblast Growth Factor Receptor Inhibitor, in Patients with Advanced or Refractory Solid Tumors. Clin. Cancer Res. Off. J. Am. Assoc. Cancer Res..

[55] Soria J.-C., Strickler J.H., Govindan R., Chai S., Chan N., Quiroga-Garcia V., Bahleda R., Hierro C., Zhong B., Gonzalez M. (2017). Safety and activity of the pan-fibroblast growth factor receptor (FGFR) inhibitor erdafitinib in phase 1 study patients (Pts) with molecularly selected advanced cholangiocarcinoma (CCA). J. Clin. Oncol..

[56] Goyal L., Bahleda R., Furuse J., Valle J.W., Moehler M.H., Oh D.-Y., Chang H.-M., Kelley R.K., Javle M.M., Borad M.J. (2019). FOENIX-101: A phase II trial of TAS-120 in patients with intrahepatic cholangiocarcinoma harboring FGFR2 gene rearrangements. J. Clin. Oncol..

[57] Borad M.J., Bridgewater J.A., Morizane C., Shroff R.T., Oh D.-Y., Moehler M.H., Furuse J., Benhadji K.A., He H., Valle J.W. (2020). A phase III study of futibatinib (TAS-120) versus gemcitabine-cisplatin (gem-cis) chemotherapy as first-line (1L) treatment for patients (pts) with advanced (adv) cholangiocarcinoma (CCA) harboring fibroblast growth factor receptor 2 (FGFR2) gene rearrangements (FOENIX-CCA3). J. Clin. Oncol..

[58] Mazzaferro V., El-Rayes B.F., Busset M.D.D., Cotsoglou C., Harris W.P., Damjanov N., Masi G., Rimassa L., Personeni N., Braiteh F. (2019). Derazantinib (ARQ 087) in advanced or inoperable FGFR2 gene fusion-positive intrahepatic cholangiocarcinoma. Br. J. Cancer.

[59] Busset M.D.D., Braun S., El-Rayes B., Harris W., Damjanov N., Masi G., Rimassa L., Bhoori S., Niger M., Personeni N. (2019). Efficacy of derazantinib (DZB) in patients (pts) with intrahepatic cholangiocarcinoma (iCCA) expressing FGFR2-fusion or FGFR2 mutations/amplifications. Ann. Oncol..

[60] Javle M.M., Abou-Alfa G.K., Macarulla T., Personeni N., Adeva J., Bergamo F., Malka D., Vogel A., Knox J.J., Evans T.R.J. (2022). Efficacy of derazantinib in intrahepatic cholangiocarcinoma patients with FGFR2 mutations or amplifications: Interim results from the phase 2 study FIDES-01. J. Clin. Oncol..

[61] Javle M.M., Roychowdhury S., Kelley R.K., Sadeghi S., Macarulla T., Waldschmidt D.T., Goyal L., Borbath I., El-Khoueiry A.B., Yong W.-P. (2021). Final results from a phase II study of infigratinib (BGJ398), an FGFR-selective tyrosine kinase inhibitor, in patients with previously treated advanced cholangiocarcinoma harboring an FGFR2 gene fusion or rearrangement. J. Clin. Oncol..

[62] Hoy S.M. (2020). Pemigatinib: First Approval. Drugs.

[63] Kang C. (2021). Infigratinib: First Approval. Drugs.

[64] Javle M., Roychowdhury S., Kelley R.K., Sadeghi S., Macarulla T., Weiss K.H., Waldschmidt D.-T., Goyal L., Borbath I., El-Khoueiry A. (2021). Infigratinib (BGJ398) in previously treated patients with advanced or metastatic cholangiocarcinoma with FGFR2 fusions or rearrangements: Mature results from a multicentre, open-label, single-arm, phase 2 study. Lancet Gastroenterol. Hepatol..

[65] Vogel A., Sahai V., Hollebecque A., Vaccaro G., Melisi D., Al-Rajabi R., Paulson A., Borad M., Gallinson D., Murphy A. (2019). FIGHT-202: A phase II study of pemigatinib in patients (pts) with previously treated locally advanced or metastatic cholangiocarcinoma (CCA). Ann. Oncol..

[66] Iqbal N., Iqbal N. (2014). Human epidermal growth factor receptor 2 (HER2) in cancers: Overexpression and therapeutic implications. Mol. Biol. Int..

[67] Wieduwilt M.J., Moasser M.M. (2008). The epidermal growth factor receptor family: Biology driving targeted therapeutics. CMLS.

[68] Yoshikawa D., Ojima H., Iwasaki M., Hiraoka N., Kosuge T., Kasai S., Hirohashi S., Shibata T. (2007). Clinicopathological and prognostic significance of EGFR, VEGF, and HER2 expression in cholangiocarcinoma. Br. J. Cancer.

[69] Hoffmann A.-C., Goekkurt E., Danenberg P.V., Lehmann S., Ehninger G., Aust D.E., Stoehlmacher-Williams J. (2013). EGFR, FLT1 and Heparanase as Markers Identifying Patients at Risk of Short Survival in Cholangiocarcinoma. PLoS ONE.

[70] Vivaldi C., Fornaro L., Ugolini C., Niccoli C., Musettini G., Pecora I., Insilla A.C., Salani F., Pasquini G., Catanese S. (2020). HER2 Overexpression as a Poor Prognostic Determinant in Resected Biliary Tract Cancer. Oncology.

[71] Javle M., Churi C., Kang H., Shroff R.T., Janku F., Surapaneni R., Zuo M., Barrera C., Alshamsi H.O., Krishnan S. (2015). HER2/neu-directed therapy for biliary tract cancer. J. Hematol. Oncol..

[72] Leone F., Cavalloni G., Pignochino Y., Sarotto I., Ferraris R., Piacibello W., Venesio T., Capussotti L., Risio M., Aglietta M. (2006). Somatic Mutations of Epidermal Growth Factor Receptor in Bile Duct and Gallbladder Carcinoma. Clin. Cancer Res. Off. J. Am. Assoc. Cancer Res..

[73] Chang Y.-T., Chang M.-C., Huang K.-W., Tung C.-C., Hsu C., Wong J.-M. (2014). Clinicopathological and prognostic significances of EGFR, KRAS and BRAF mutations in biliary tract carcinomas in Taiwan. J. Gastroenterol. Hepatol..

[74] Gwak G.-Y., Yoon J.-H., Shin C.M., Ahn Y.J., Chung J.K., Kim Y.A., Kim T.-Y., Lee H.-S. (2005). Detection of response-predicting mutations in the kinase domain of the epidermal growth factor receptor gene in cholangiocarcinomas. J. Cancer Res. Clin. Oncol..

[75] Lynch T.J., Bell D.W., Sordella R., Gurubhagavatula S., Okimoto R.A., Brannigan B.W., Harris P.L., Haserlat S.M., Supko J.G., Haluska F.G. (2004). Activating mutations in the epidermal growth factor receptor underlying responsiveness of non-small-cell lung cancer to gefitinib. N. Engl. J. Med..

[76] Bokemeyer C., Van Cutsem E., Rougier P., Ciardiello F., Heeger S., Schlichting M., Celik I., Köhne C.-H. (2012). Addition of cetuximab to chemotherapy as first-line treatment for KRAS wild-type metastatic colorectal cancer: Pooled analysis of the CRYSTAL and OPUS randomised clinical trials. Eur. J. Cancer.

[77] Slamon D.J., Leyland-Jones B., Shak S., Fuchs H., Paton V., Bajamonde A., Fleming T., Eiermann W., Wolter J., Pegram M. (2001). Use of Chemotherapy plus a Monoclonal Antibody against HER2 for Metastatic Breast Cancer That Overexpresses HER. N. Engl. J. Med..

[78] Bang Y.-J., Van Cutsem E., Feyereislova A., Chung H.C., Shen L., Sawaki A., Lordick F., Ohtsu A., Omuro Y., Satoh T. (2010). Trastuzumab in combination with chemotherapy versus chemotherapy alone for treatment of HER2-positive advanced gastric or gastro-oesophageal junction cancer (ToGA): A phase 3, open-label, randomised controlled trial. Lancet.

[79] Lee J., Park S.H., Chang H.M., Kim J.S., Choi H.J., Lee M.A., Chang J.S., Jeung H.C., Kang J.H., Lee H.W. (2012). Gemcitabine and oxaliplatin with or without erlotinib in advanced biliary-tract cancer: A multicentre, open-label, randomised, phase 3 study. Lancet Oncol..

[80] Gruenberger B., Schueller J., Heubrandtner U., Wrba F., Tamandl D., Kaczirek K., Roka R., Freimann-Pircher S., Gruenberger T. (2010). Cetuximab, gemcitabine, and oxaliplatin in patients with unresectable advanced or metastatic biliary tract cancer: A phase 2 study. Lancet Oncol..

[81] Malka D., Cervera P., Foulon S., Trarbach T., de la Fouchardière C., Boucher E., Fartoux L., Faivre S., Blanc J.-F., Viret F. (2014). Gemcitabine and oxaliplatin with or without cetuximab in advanced biliary-tract cancer (BINGO): A randomised, open-label, non-comparative phase 2 trial. Lancet Oncol..

[82] Leone F., Marino D., Cereda S., Filippi R., Belli C., Spadi R., Nasti G., Montano M., Amatu A., Aprile G. (2015). Panitumumab in combination with gemcitabine and oxaliplatin does not prolong survival in wild-typeKRASadvanced biliary tract cancer: A randomized phase 2 trial (Vecti-BIL study). Cancer.

[83] Vogel A., Kasper S., Bitzer M., Block A., Sinn M., Schulze-Bergkamen H., Moehler M., Pfarr N., Endris V., Goeppert B. (2018). PICCA study: Panitumumab in combination with cisplatin/gemcitabine chemotherapy in KRAS wild-type patients with biliary cancer—A randomised biomarker-driven clinical phase II AIO study. Eur. J. Cancer.

[84] Peck J., Wei L., Zalupski M., O’Neil B., Calero M.V., Bekaii-Saab T. (2012). HER2/neu May Not Be an Interesting Target in Biliary Cancers: Results of an Early Phase II Study with Lapatinib. Oncology.

[85] Ramanathan R.K., Belani C., Singh D.A., Tanaka M., Lenz H.-J., Yen Y., Kindler H.L., Iqbal S., Longmate J., Mack P.C. (2009). A phase II study of lapatinib in patients with advanced biliary tree and hepatocellular cancer. Cancer Chemother. Pharmacol..

[86] Mondaca S., Razavi P., Xu C., Offin M., Myers M., Scaltriti M., Hechtman J.F., Bradley M., O’Reilly E.M., Berger M.F. (2019). Genomic Characterization of ERBB2-Driven Biliary Cancer and a Case of Response to Ado-Trastuzumab Emtansine. JCO Precis. Oncol..

[87] Harding J.J., Cleary J.M., Quinn D.I., Braña I., Moreno V., Borad M.J., Loi S., Spanggaard I., Park H., Ford J.M. (2021). Targeting HER2 (ERBB2) mutation-positive advanced biliary tract cancers with neratinib: Results from the phase II SUMMIT ‘basket’ trial. J. Clin. Oncol..

[88] Roa I., de Toro G., Schalper K., de Aretxabala X., Churi C., Javle M. (2014). Overexpression of the HER2/Neu Gene: A New Therapeutic Possibility for Patients with Advanced Gallbladder Cancer. Gastrointest. Cancer Res..

[89] Li K., Luo H., Huang L., Luo H., Zhu X. (2020). Microsatellite instability: A review of what the oncologist should know. Cancer Cell Int..

[90] Dudley J.C., Lin M.-T., Le D.T., Eshleman J.R. (2016). Microsatellite Instability as a Biomarker for PD-1 Blockade. Clin. Cancer Res. Off. J. Am. Assoc. Cancer Res..

[91] Suto T., Habano W., Sugai T., Uesugi N., Kanno S., Saito K., Nakamura S.I. (2001). Infrequent Microsatellite Instability in Biliary Tract Cancer. J. Surg. Oncol..

[92] Winkelmann R., Schneider M., Hartmann S., Schnitzbauer A.A., Zeuzem S., Peveling-Oberhag J., Hansmann M.L., Walter D. (2018). Microsatellite Instability Occurs Rarely in Patients with Cholangiocarcinoma: A Retrospective Study from a German Tertiary Care Hospital. Int. J. Mol. Sci..

[93] Ju J.Y., Dibbern M.E., Mahadevan M.S., Fan J., Kunk P.R., Stelow E.B. (2019). Mismatch Repair Protein Deficiency/Microsatellite Instability Is Rare in Cholangiocarcinomas and Associated with Distinctive Morphologies. Am. J. Clin. Pathol..

[94] Ikeda Y., Ono M., Ohmori G., Ameda S., Yamada M., Abe T., Fujii S., Fujita M., Maeda M. (2021). Successful pembrolizumab treatment of microsatellite instability-high intrahepatic cholangiocarcinoma: A case report. Clin. Case Rep..

[95] Czink E., Kloor M., Goeppert B., Fröhling S., Uhrig S., Weber T.F., Meinel J., Sutter C., Weiss K.H., Schirmacher P. (2017). Successful immune checkpoint blockade in a patient with advanced stage microsatellite-unstable biliary tract cancer. Cold Spring Harbor Mol. Case Stud..

[96] Nakamura M., Ueno M., Hayami S., Kawai M., Miyamoto A., Suzaki N., Hirono S., Okada K.-I., Miyazawa M., Kitahata Y. (2020). Effective Response of Intrahepatic Cholangiocarcinoma to Pembrolizumab: A Case Report. Anticancer Res..

[97] Naganuma A., Sakuda T., Murakami T., Aihara K., Watanuki Y., Suzuki Y., Shibasaki E., Masuda T., Uehara S., Yasuoka H. (2020). Microsatellite Instability-high Intrahepatic Cholangiocarcinoma with Portal Vein Tumor Thrombosis Successfully Treated with Pembrolizumab. Intern. Med..

[98] Lee S.H., Lee H.S., Lee S.H., Woo S.M., Kim D.U., Bang S. (2020). Efficacy and Safety of Pembrolizumab for Gemcitabine/Cisplatin-Refractory Biliary Tract Cancer: A Multicenter Retrospective Study. J. Clin. Med..

[99] Kang J., Jeong J.H., Hwang H.-S., Lee S.S., Park D.H., Oh D.W., Song T.J., Kim K.-H., Hwang S., Hwang D.W. (2020). Efficacy and Safety of Pembrolizumab in Patients with Refractory Advanced Biliary Tract Cancer: Tumor Proportion Score as a Potential Biomarker for Response. Cancer Res. Treat..

[100] Marcus L., Lemery S.J., Keegan P., Pazdur R. (2019). FDA Approval Summary: Pembrolizumab for the Treatment of Microsatellite Instability-High Solid Tumors. Clin. Cancer Res. Off. J. Am. Assoc. Cancer Res..

[101] Hanahan D., Weinberg R.A. (2011). Hallmarks of cancer: The next generation. Cell.

[102] Thelen A., Scholz A., Benckert C., Schröder M., Weichert W., Wiedenmann B., Neuhaus P., Jonas S. (2008). Microvessel density correlates with lymph node metastases and prognosis in hilar cholangiocarcinoma. J. Gastroenterol..

[103] Chen Y., Chen Y., Yu G., Ding H. (2011). Lymphangiogenic and Angiogentic Microvessel Density in Gallbladder Carcinoma. Hepato-Gastroenterology.

[104] Thelen A., Scholz A., Weichert W., Wiedenmann B., Neuhaus P., Geßner R., Benckert C., Jonas S. (2010). Tumor-Associated Angiogenesis and Lymphangiogenesis Correlate with Progression of Intrahepatic Cholangiocarcinoma. Am. J. Gastroenterol..

[105] Guedj N., Zhan Q., Perigny M., Rautou P.-E., Degos F., Belghiti J., Farges O., Bedossa P., Paradis V. (2009). Comparative protein expression profiles of hilar and peripheral hepatic cholangiocarcinomas. J. Hepatol..

[106] Grimaldi A., Guida T., D’Attino R., Perrotta E., Otero M., Masala A., Cartenì G. (2007). Sunitinib: Bridging present and future cancer treatment. Ann. Oncol. Off. J. Eur. Soc. Med. Oncol..

[107] Marisi G., Cucchetti A., Ulivi P., Canale M., Cabibbo G., Solaini L., Foschi F.G., De Matteis S., Ercolani G., Valgiusti M. (2018). Ten years of sorafenib in hepatocellular carcinoma: Are there any predictive and/or prognostic markers?. World J. Gastroenterol..

[108] Abdelgalil A.A., Alkahtani H.M., Al-Jenoobi F.I. (2019). Sorafenib. Profiles Drug Substan. Excip. Relat. Methodol..

[109] Ettrich T.J., Seufferlein T. (2018). Regorafenib. Recent results in cancer research. Fortschritte der Krebsforschung. Prog. Recher. Cancer.

[110] Yi J.H., Thongprasert S., Lee J., Doval D., Park S.H., Park J.O., Park Y.S., Kang W.K., Lim H.Y. (2012). A phase II study of sunitinib as a second-line treatment in advanced biliary tract carcinoma: A multicentre, multinational study. Eur. J. Cancer.

[111] Viallard C., Larrivée B. (2017). Tumor angiogenesis and vascular normalization: Alternative therapeutic targets. Angiogenesis.

[112] Valle J.W., Vogel A., Denlinger C.S., He A.R., Bai L.-Y., Orlova R., Van Cutsem E., Adeva J., Chen L.-T., Obermannova R. (2021). Addition of ramucirumab or merestinib to standard first-line chemotherapy for locally advanced or metastatic biliary tract cancer: A randomised, double-blind, multicentre, phase 2 study. Lancet Oncol..

[113] Taylor M.H., Schmidt E.V., Dutcus C., Pinheiro E.M., Funahashi Y., Lubiniecki G., Rasco D. (2021). The LEAP program: Lenvatinib plus pembrolizumab for the treatment of advanced solid tumors. Futur. Oncol..

[114] Bengala C., Bertolini F., Malavasi N., Boni C., Aitini E., Dealis C., Zironi S., Depenni R., Fontana A., Del Giovane C. (2009). Sorafenib in patients with advanced biliary tract carcinoma: A phase II trial. Br. J. Cancer.

[115] El-Khoueiry A.B., Rankin C.J., Ben-Josef E., Lenz H.-J., Gold P.J., Hamilton R.D., Govindarajan R., Eng C., Blanke C.D. (2011). SWOG 0514: A phase II study of sorafenib in patients with unresectable or metastatic gallbladder carcinoma and cholangiocarcinoma. Investig. New Drugs.

[116] Sun W., Patel A., Normolle D., Patel K., Ohr J., Lee J.J., Bahary N., Chu E., Streeter N., Drummond S. (2018). A phase 2 trial of regorafenib as a single agent in patients with chemotherapy-refractory, advanced, and metastatic biliary tract adenocarcinoma. Cancer.

[117] Demols A., Borbath I., Van den Eynde M., Houbiers G., Peeters M., Marechal R., Delaunoit T., Goemine J.-C., Laurent S., Holbrechts S. (2020). Regorafenib after failure of gemcitabine and platinum-based chemotherapy for locally advanced/metastatic biliary tumors: REACHIN, a randomized, double-blind, phase II trial. Ann. Oncol. Off. J. Eur. Soc. Med. Oncol..

[118] Ueno M., Ikeda M., Sasaki T., Nagashima F., Mizuno N., Shimizu S., Ikezawa H., Hayata N., Nakajima R., Morizane C. (2020). Phase 2 study of lenvatinib monotherapy as second-line treatment in unresectable biliary tract cancer: Primary analysis results. BMC Cancer.

[119] Zhu A.X., Meyerhardt J.A., Blaszkowsky L.S., Kambadakone A.R., Muzikansky A., Zheng H., Clark J.W., Abrams T.A., Chan J.A., Enzinger P.C. (2010). Efficacy and safety of gemcitabine, oxaliplatin, and bevacizumab in advanced biliary-tract cancers and correlation of changes in 18-fluorodeoxyglucose PET with clinical outcome: A phase 2 study. Lancet Oncol..

[120] Iyer R.V., Pokuri V.K., Groman A., Ma W.W., Malhotra U., Iancu D.M., Grande C., Saab T.B. (2018). A Multicenter Phase II Study of Gemcitabine, Capecitabine, and Bevacizumab for Locally Advanced or Metastatic Biliary Tract Cancer. Am. J. Clin. Oncol..

[121] Lee J.K., Capanu M., O’Reilly E.M., Ma J., Chou J.F., Shia J., Katz S.S., Gansukh B., Reidylagunes D., Segal N.H. (2013). A phase II study of gemcitabine and cisplatin plus sorafenib in patients with advanced biliary adenocarcinomas. Br. J. Cancer.

[122] Moehler M., Maderer A., Schimanski C., Kanzler S., Denzer U., Kolligs F., Ebert M., Distelrath A., Geissler M., Trojan J. (2014). Gemcitabine plus sorafenib versus gemcitabine alone in advanced biliary tract cancer: A double-blind placebo-controlled multicentre phase II AIO study with biomarker and serum programme. Eur. J. Cancer.

[123] Santoro A., Gebbia V., Pressiani T., Testa A., Personeni N., Arrivas Bajardi E., Foa P., Buonadonna A., Bencardino K., Barone C. (2014). A randomized, multicenter, phase II study of vandetanib monotherapy versus vandetanib in combination with gemcitabine versus gemcitabine plus placebo in subjects with advanced biliary tract cancer: The VanGogh study. Ann. Oncol. Off. J. Eur. Soc. Med. Oncol..

[124] Jensen L.H., Fernebro E., Ploen J., Eberhard J., Lindebjerg J., Jakobsen A. (2015). Randomized phase II crossover trial exploring the clinical benefit from targeting EGFR or VEGF with combination chemotherapy in patients with non-resectable biliary tract cancer. J. Clin. Oncol..

[125] Valle J.W., Wasan H., Lopes A., Backen A.C., Palmer D.H., Morris K., Duggan M., Cunningham D., Anthoney D.A., Corrie P. (2015). Cediranib or placebo in combination with cisplatin and gemcitabine chemotherapy for patients with advanced biliary tract cancer (ABC-03): A randomised phase 2 trial. Lancet Oncol..

[126] Lubner S.J., Mahoney M.R., Kolesar J.L., LoConte N.K., Kim G.P., Pitot H.C., Philip P.A., Picus J., Yong W.-P., Horvath L. (2010). Report of a Multicenter Phase II Trial Testing a Combination of Biweekly Bevacizumab and Daily Erlotinib in Patients with Unresectable Biliary Cancer: A Phase II Consortium Study. J. Clin. Oncol. Off. J. Am. Soc. Clin. Oncol..

[127] El-Khoueiry A.B., Rankin C., Siegel A.B., Iqbal S., Gong I.-Y., Micetich K., Kayaleh O.R., Lenz H.-J., Blanke C.D. (2014). S0941: A phase 2 SWOG study of sorafenib and erlotinib in patients with advanced gallbladder carcinoma or cholangiocarcinoma. Br. J. Cancer.

[128] Lin J., Yang X., Long J., Zhao S., Mao J., Wang D., Bai Y., Bian J., Zhang L., Yang X. (2020). Pembrolizumab combined with lenvatinib as non-first-line therapy in patients with refractory biliary tract carcinoma. Hepatobiliary Surg. Nutr..

[129] Santarpia L., Lippman S.M., El-Naggar A.K. (2012). Targeting the MAPK–RAS–RAF signaling pathway in cancer therapy. Expert Opin. Ther. Targets.

[130] Moore A.R., Rosenberg S.C., McCormick F., Malek S. (2020). RAS-targeted therapies: Is the undruggable drugged?. Nat. Rev. Drug Discov..

[131] Skoulidis F., Li B.T., Dy G.K., Price T.J., Falchook G.S., Wolf J., Italiano A., Schuler M., Borghaei H., Barlesi F. (2021). Sotorasib for Lung Cancers with KRAS p.G12C Mutation. N. Engl. J. Med..

[132] Zhang S.S., Nagasaka M. (2021). Spotlight on Sotorasib (AMG 510) for KRASG12C Positive Non-Small Cell Lung Cancer. Lung Cancer.

[133] Bekaii-Saab T.S., Spira A.I., Yaeger R., Buchschacher G.L., McRee A.J., Sabari J.K., Johnson M.L., Barve M.A., Hafez N., Velastegui K. (2022). KRYSTAL-1: Updated activity and safety of adagrasib (MRTX849) in patients (Pts) with unresectable or metastatic pancreatic cancer (PDAC) and other gastrointestinal (GI) tumors harboring a KRASG12C mutation. J. Clin. Oncol..

[134] Li W., Cui Y., Yin F., Peng L., Liu X., Shen Y., Guo Y., Wen S., Shi J., Lei M. (2020). BRAF mutation in Chinese biliary tract cancer patients. J. Clin. Oncol..

[135] Tannapfel A., Sommerer F., Benicke M., Katalinic A., Uhlmann D., Witzigmann H., Hauss J., Wittekind C. (2003). Mutations of the BRAF gene in cholangiocarcinoma but not in hepatocellular carcinoma. Gut.

[136] Robertson S., Hyder O., Dodson R., Nayar S.K., Poling J., Beierl K., Eshleman J.R., Lin M.-T., Pawlik T.M., Anders R.A. (2013). The frequency of KRAS and BRAF mutations in intrahepatic cholangiocarcinomas and their correlation with clinical outcome. Hum. Pathol..

[137] Hyman D.M., Puzanov I., Subbiah V., Faris J.E., Chau I., Blay J.-Y., Wolf J.L., Raje N.S., Diamond E., Hollebecque A. (2015). Vemurafenib in Multiple Nonmelanoma Cancers with BRAF V600 Mutations. N. Engl. J. Med..

[138] Flaherty K.T., Infante J.R., Daud A., Gonzalez R., Kefford R.F., Sosman J., Hamid O., Schuchter L., Cebon J., Ibrahim N. (2012). Combined BRAF and MEK Inhibition in Melanoma with BRAF V600 Mutations. N. Engl. J. Med..

[139] Abraham J., Stenger M. (2014). Dabrafenib in advanced melanoma with BRAF V600E mutation. J. Commun. Support. Oncol..

[140] Bekaii-Saab T., Phelps M.A., Li X., Saji M., Goff L., Kauh J.S.W., O’Neil B.H., Balsom S., Balint C., Liersemann R. (2011). Multi-Institutional Phase II Study of Selumetinib in Patients with Metastatic Biliary Cancers. J. Clin. Oncol. Off. J. Am. Soc. Clin. Oncol..

[141] Bridgewater J., Lopes A., Beare S., Duggan M., Lee D., Ricamara M., McEntee D., Sukumaran A., Wasan H., Valle J.W. (2016). A phase 1b study of Selumetinib in combination with Cisplatin and Gemcitabine in advanced or metastatic biliary tract cancer: The ABC-04 study. BMC Cancer.

[142] Doherty M., Tam V.C., McNamara M.G., Hedley D.W., Dhani N.C., Chen E.X., Jang R.W.-J., Tang P.A., Sim H.-W., O’Kane G.M. (2018). Selumetinib (Sel) and cisplatin/gemcitabine (CisGem) for advanced biliary tract cancer (BTC): A randomized trial. J. Clin. Oncol..

[143] Sullivan R.J., Infante J.R., Janku F., Wong D.J.L., Sosman J.A., Keedy V., Patel M.R., Shapiro G.I., Mier J.W., Tolcher A.W. (2018). First-in-Class ERK1/2 Inhibitor Ulixertinib (BVD-523) in Patients with MAPK Mutant Advanced Solid Tumors: Results of a Phase I Dose-Escalation and Expansion Study. Cancer Discov..

[144] Lord C.J., Ashworth A. (2017). PARP inhibitors: Synthetic lethality in the clinic. Science.

[145] Sulkowski P.L., Corso C.D., Robinson N.D., Scanlon S.E., Purshouse K.R., Bai H., Liu Y., Sundaram R.K., Hegan D.C., Fons N.R. (2017). 2-Hydroxyglutarate produced by neomorphic IDH mutations suppresses homologous recombination and induces PARP inhibitor sensitivity. Sci. Transl. Med..

[146] Herberger B., Puhalla H., Lehnert M., Wrba F., Novak S., Brandstetter A., Gruenberger B., Gruenberger T., Pirker R., Filipits M. (2007). Activated Mammalian Target of Rapamycin Is an Adverse Prognostic Factor in Patients with Biliary Tract Adenocarcinoma. Clin. Cancer Res. Off. J. Am. Assoc. Cancer Res..

[147] Hansel D.E., Rahman A., Hidalgo M., Thuluvath P.J., Lillemoe K.D., Schulick R., Ku J.-L., Park J.-G., Miyazaki K., Ashfaq R. (2003). Identification of Novel Cellular Targets in Biliary Tract Cancers Using Global Gene Expression Technology. Am. J. Pathol..

[148] Riener M.-O., Bawohl M., Clavien P.-A., Jochum W. (2008). RarePIK3CA hotspot mutations in carcinomas of the biliary tract. Genes Chromosom. Cancer.

[149] Buzzoni R., Pusceddu S., Bajetta E., de Braud F.G.M., Platania M., Iannacone C., Cantore M., Mambrini A., Bertolini A., Alabiso O. (2014). Activity and safety of RAD001 (everolimus) in patients affected by biliary tract cancer progressing after prior chemotherapy: A phase II ITMO study. Ann. Oncol. Off. J. Eur. Soc. Med. Oncol..

[150] Lau D.K., Tay R.Y., Yeung Y.H., Chionh F., Mooi J., Murone C., Skrinos E., Price T.J., Mariadason J.M., Tebbutt N.C. (2018). Phase II study of everolimus (RAD001) monotherapy as first-line treatment in advanced biliary tract cancer with biomarker exploration: The RADiChol Study. Br. J. Cancer.

[151] Tan E.S., Cao B., Kim J., Al-Toubah T.E., Mehta R., Centeno B.A., Kim R.D. (2020). Phase 2 study of copanlisib in combination with gemcitabine and cisplatin in advanced biliary tract cancers. Cancer.

[152] Vaishnavi A., Le A.T., Doebele R.C. (2014). TRKing Down an Old Oncogene in a New Era of Targeted Therapy. Cancer Discov..

[153] Cocco E., Scaltriti M., Drilon A. (2018). NTRK fusion-positive cancers and TRK inhibitor therapy. Nat. Rev. Clin. Oncol..

[154] Gu T.-L., Deng X., Huang F., Tucker M., Crosby K., Rimkunas V., Wang Y., Deng G., Zhu L., Tan Z.-P. (2011). Survey of Tyrosine Kinase Signaling Reveals ROS Kinase Fusions in Human Cholangiocarcinoma. PLoS ONE.

[155] Doebele R.C., Drilon A., Paz-Ares L., Siena S., Shaw A.T., Farago A.F., Blakely C.M., Seto T., Cho B.C., Tosi D. (2020). Entrectinib in patients with advanced or metastatic NTRK fusion-positive solid tumours: Integrated analysis of three phase 1–2 trials. Lancet Oncol..

[156] Drilon A., Laetsch T.W., Kummar S., Dubois S.G., Lassen U.N., Demetri G.D., Nathenson M., Doebele R.C., Farago A.F., Pappo A.S. (2018). Efficacy of Larotrectinib in *TRK* Fusion–Positive Cancers in Adults and Children. N. Engl. J. Med..

